# Investigation and control of a *Plasmodium falciparum* malaria outbreak in Shan Special Region II of Myanmar along the China-Myanmar Border from June to December 2014

**DOI:** 10.1186/s40249-016-0127-8

**Published:** 2016-04-25

**Authors:** Hui Liu, Jian-Wei Xu, Heng-Lin Yang, Mei Li, Cheng-De Sun, Yi-Jie Yin, Zhi-Liang Zheng, Guang-Yun Zhang, Ai-Shui Yu, Yong-Hui Yang, Chun-Hui Li, Shui Ai

**Affiliations:** Yunnan Institute of Parasitic Diseases, Yunnan Provincial Center of Malaria Research, Yunnan Provincial Collaborative Innovation Center for Public Health and Disease Prevention and Control, Yunnan Provincial Key Laboratory of Vector-borne Diseases Control and Research, Puer, 665000 China; National Institute of Parasitic Diseases, Chinese Center for Disease Control and Prevention, Shanghai, 200025 China; Wa State Department of Health, Pangsang, Shan Special Region II Myanmar; Wa State Office of Health Poverty Action, Pangsang, Shan Special Region II Myanmar; Menglian Center for Disease Control and Prevention, Menglian, 665800 China; Wa State Center for Disease Control and Prevention, Pangsang, Shan Special Region II Myanmar

**Keywords:** Malaria outbreak, *Plasmodium falciparum*, Investigation, Control, Chinese-Myanmar border

## Abstract

**Background:**

From 2007 to 2013, intensive control measures reduced malaria burden by 90 % along the China-Myanmar border. However, despite these measures a *P. falciparum* malaria outbreak was reported in the Shan Special Region II of Myanmar in June of 2014.

**Methods:**

Epidemiological, parasitological and entomological investigations were performed. Dihydroartemisinin piperaquine (DAPQ) was immediately administered to treat parasite positive individuals. Long lasting insecticidal nets (LLIN), indoor residual spraying (IRS) with insecticides and behavior change communication (BCC) were also provided for outbreak control. An embedded efficacy study was conducted evaluating DP. Molecular genotyping via polymerase chain reaction (PCR) was performed on the Kelch gene on chromosome 13.

**Results:**

All infections were identified as *Plasmodium falciparum* by RDT and microscopy. Two fatalities resulted from the outbreak. The attack rate was 72.8 % (67/92) and the incidence density rate was 14.2 per 100 person-weeks. The positive rate of rapid diagnostic test (RDT) was 72.2 % (65/90) and microscopically-determine parasite rate 42.2 % (38/90). Adjusted odds ratio (*OR*) of multivariate logistic regression analysis for aged <15 years, 15–45 years, inappropriate treatment from a private healer and lack of bed nets were 13.51 (95 % confidence interval, 2.21–105.89), 7.75 (1.48–44.97), 3.78 (1.30–46.18) and 3.21(1.21–15.19) respectively. In the six surrounding communities of the outbreak site, positive RDT rate was 1.2 % (4/328) and microscopically-determine parasite rate 0.6 % (2/328). Two light traps collected a total of 110 anopheline mosquitoes including local vectors, *An. minimus*, *An. sinensis* and *An. maculates*. After intensive control, the detection of malaria attacks, parasites and antigen were reduced to zero between July 1 and December 1, 2014. The cure rate of *P. falciparum* patients at day 42 was 94.3 % (95 % *CI*, 80.8–99.3 %). The PCR did not detect K13-propeller mutations.

**Conclusion:**

Imported *P. falciparum* caused the outbreak. Age, seeking inappropriate treatment and lack of bed nets were risk factors for infection during the outbreak. *P. falciparum* was sensitive to treatment with DAPQ. The integrated measures controlled the outbreak and prevented the spread of *P. falciparum* effectively. The results of this study indicate that malaria control on the China-Myanmar border, especially among special populations, needs further collaboration between China, Myanmar and international societies.

**Electronic supplementary material:**

The online version of this article (doi:10.1186/s40249-016-0127-8) contains supplementary material, which is available to authorized users.

## Multilingual abstracts

Please see Additional file 1 for translations of the abstract into the six official working languages of the United Nations.

## Background

Remarkable progress has been achieved in reducing malaria cases and deaths over the last decade. The malaria target under the Millennium Development Goal 6 has been met, and 55 countries are on track to reduce their malaria burden by 75 %, in line with the World Health Assembly’s target for 2015 [1]. The 34 countries have either declared a national policy for malaria elimination or are pursuing spatially progressive elimination within their borders [2, 3]. Countries in the Asia Pacific region are making substantial progress towards eliminating malaria [4], with China aiming to eliminate malaria by 2020 [5]. However, an estimated 3.2 billion people are still at risk of being infected with malaria and developing the disease, and 1.2 billion are at high risk (>1 in 1 000 chance of getting malaria in a year) across the world [1]. The Greater Mekong Subregion (GMS) consisting of Cambodia, Yunnan Province of China, Laos, Myanmar, Thailand and Vietnam, has been one of the most dangerous foci for malaria. Around 70 % of the total population of this region are still at risk of contracting malaria, with 26 % at high risk [6, 7]. Of the six countries in the GMS, Myanmar has the highest malaria burden and ranks 31st for countries with the highest malaria burden globally [6, 8]. Meanwhile, the emergence of *P. falciparum* partial resistance to artemisinin has been one of the most concerning issues [1, 9–13].

With the nascent goal for regional malaria elimination in the GMS, arises the need to develop regional and local initiatives. Malaria control along the international border and attempts to contain drug resistance have received much attention, but effective interventions remain challenging [1, 5, 9]. China has prioritized the control of cross-border transmission of malaria along the 2185 km China-Myanmar border [14]. During 2007–2013, with the support of the Global Fund to Fight AIDS, Tuberculosis and Malaria (GFATM), intensive efforts have reduced malaria burden by 95 % on the Chinese side and by 89 % in Myanmar’s border areas [15–17]; meanwhile, the drug susceptibilty of malaria parasites has not significantly changed in those areas [18, 19]. In the Shan Special Region II (locally called Wa State) of Myanmar, malaria is effectively controlled with parasite prevalence decreasing from 11.9 % in March 2008 [16] to 0 % in November 2013 [17]. However, on June 19^th^, 2014, a *P. falciparum* malaria outbreak was reported in the Aidao Rubber Plantation, located close to the border. Officials from China, Myanmar and Health Poverty Action (HPA) jointly conducted the outbreak investigation and led the control efforts. Here is reported the outbreak investigation, the public health response and the efficacy results from an integrated efficacy study along with a discussion on the implications of our findings.

## Methods

### Outbreak site

**Fig. 1:**
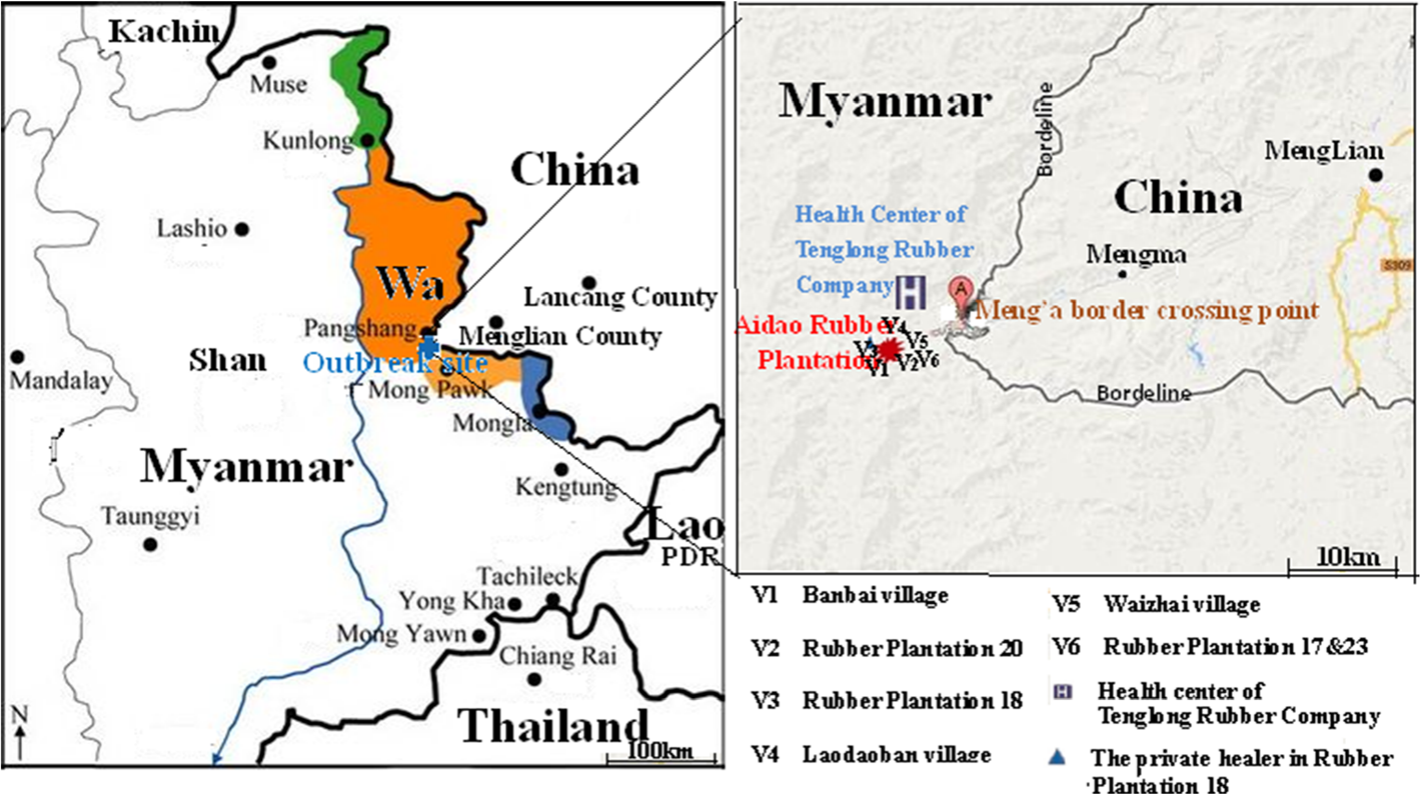
Study site relative to neighboring areas

The outbreak was reported in a private rubber plantation, Aido Rubber Plantation (ARP), about 10 km away from the China-Myanmar border. The plantation was established in 2004. At the time when the outbreak was reported, there were 122 inhabitants, in 24 households of which 14 families were of the Lahu ethnic minority who emigrated from Lancang County, China in 2004. There were 10 families of the Wa ethnic minority who emigrated from other villages of the Shan Special region of Myanmar in 2005. All houses have wood and bamboo walls and grass or asbestos roofs that enable easy entry for mosquitoes. There were only three bed nets in the whole community. The Health Center of Tenglong Rubber Company (HCTRC) is approximately 8 kilometers away from ARP and is the nearest public health facility. Additionally, there is a private healer at Rubber Plantation 18 about 2 kilometers away from ARP (Fig. 1).

### Outbreak investigation

#### Focus identification

**Fig. 2:**
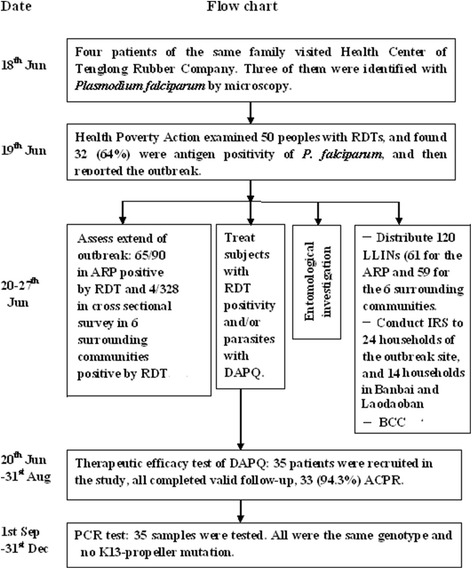
Profile for outbreak investigation, response and control in Shan Special Region II of Myanmar, June – December 2014

Four patients from the same family visited the HCTRC on June 18^th^, 2014. Three of the four were diagnosed with *P. falciparum* infection by microscopy. This was immediately reported by the HCTRC to the Wa State Office of the HPA. To better identify the focus of the outbreak, the HPA examined 50 individuals who actively requested malaria screening with rapid diagnostic test (RDT) on the morning of June 19. The results were reported to Wa State Department of Health in Myanmar; Kunming office of HPA, Yunnan Institute of Parasitic Diseases (YIPD) and Menglian Center for Disease Control and Prevention (CDC) in China (Fig. 2).

#### Estimation of outbreak size and risk factors

To estimate the size of outbreak, all inhabitants of ARP were tested by RDT (Standard Diagnostics, FK80 P.f/P.v RDT, Hagal-Dong, Korea) and axillary temperatures were measured. Additionally, thick and thin blood smears were prepared for all subjects including the 50 individuals previously tested by the HPA with RDT. A standardized questionnaire to assess risk factors was completed during a face-to-face interview with each patient or caretaker. The data were collected from each individual consisting of age, gender, number and date of malaria attacks, gravity of attacks, locations from which treatment was sought, results of RDTs and microscopy, ownership of bed nets and type of house, and travel excursions between May 1 and June 19 (Fig. 2).

In addition, a short cross-sectional survey was conducted from June 21^st^ to 26^th^, 2014 in all six surrounding communities ranging between 1 and 5 km from the ARP. The six communities were Bangbai village, Rubber Plantation 20, Rubber Plantation 18, Laodaoban village, Waizhai village and Rubber Plantation 17 & 23 (Fig. 1). During the community visits, all inhabitants who were at home and agreed to take part in the survey were tested by both RDT and microscopy. The questionnaire was not conducted in these six communities.

#### Retrospective data collection

Retrospective data was collected to identify the index case and to acquire contextual knowledge around the outbreak by reviewing HCTRC records and using non-directive interviews with private healers and patients or caretakers who also participated in the standardized questionnaires.

### Outbreak response and control

#### Diagnosis and case definition

Any patient with a positive RDT was defined as a malaria case for treatment.

#### Treatment of cases

DAPQ (40 mg base dihydroartemisinin and 320 mg piperaquine phosphate per tablet) was administered to every malaria case, once a day for 3 days [19]. Patients recruited into the embedded efficacy study were given directly observed therapy. Other cases were not supervised.

#### Other outbreak control measures

On June 20, 2014, a total of 61 long lasting insecticidal nets (LLIN) were distributed to each household in the outbreak site to isolate malaria patients from uninfected inhabitants. On the next day, an extra 59 LLINs were distributed to the six other communities to replace torn nets. Indoor residual spraying (IRS) with lambda-cyhalothrin (Syngenta, Jiangsu, China) was carried out at a dose of 25 mg per m^2^ to all houses of the ARP, and houses of malaria patients and their neighbors in the six surrounding communities on June 27^th^. Two community health workers (CHW) were trained and equipped with RDT and DP to carry out diagnosis and treatment of malaria. At least one CHW visited the ARP and its surrounding villages each day from June 21^st^ to August 31^st^, to actively detect and treat malaria patients. Behavior change communication (BCC) was conducted from June 20 to 27, 2014. Investigators communicated with every patient or caretaker while they administered a face-to-face interview, and the CHWs also conducted BCC during their household visits. The core information of communication included the cause of malaria, vectors, high risk areas, seeking appropriate diagnosis and treatment, and instruction on the use of bed nets (Fig. 2). Intensified monitoring by microscopy for suspected cases continued at HCTRC until 31 December, 2014.

### Entomological survey

At the outbreak site, two light traps (Photocatalysis type, Kung Fu Xiaoshuai, Wuhan JiXing, China) were used to collect both indoor and outdoor mosquitoes overnight. The genus was identified in the field, and anophelines were sent to laboratory of Menglian CDC for identification of species [20].

### Embedded efficacy study

#### Patients and recruiting criteria

Based on the results of microscopy, patients with mono-infections of *P. falciparum,* older than 1 year, with a body weight ≥ 5 kg, and presenting with a parasite density of 500–100 000 parasites per μL, were recruited to this study, after obtaining full informed consent. Exclusion criteria were defined as previously described [21].

#### Study design

The study design was based on the standard 42 day follow up survey as defined by the WHO [21, 22]. The two trained CHWs visited patient homes on day 1, 2 and 3 to give directly observed treatment. Patients were then assessed at follow up visits on days 7, 14, 21, 28, 35 and 42 or whenever they had signs and symptoms consistent with malaria.

#### Treatment

DAPQ was sourced from Zhejiang Holley Nanhu Pharmaceutical Co. Ltd, and provided at 65 mg/kg body weight.

#### Clinical procedures

A general physical exam was conducted at enrollment. Axillary temperature was measured and blood was collected for blood smears and filter paper spots (about 100uL blood per patient) at enrollment, every 8–12 h in the first 3 days and then on day 7, 14, 21, 28, 35 and 42.

#### Laboratory investigation

Malaria blood films were stained with Giemsa, and slides were examined by two independent microscopists in YIPD and considered negative if no parasites were seen after examination of 200 oil-immersion fields in a thick blood film. Parasite clearance was defined as no detection of asexual parasites per 500 white blood cells in two sequential microscopic examinations within an 8–12 h interval [19]. PCR was performed in the National Institute of Parasitic Diseases, China CDC to distinguish between reinfection and recurrence of blood stage infections by genotyping and to confirm the *Plasmodium* species or, if needed, to detect mixed infections. DNA extraction and genotype analysis were conducted based on investigation of the three polymorphic genetic markers *msp1*, *msp2*, and *glurp*, according to WHO recommended procedures [19, 23, 24]. Recrudescence was defined as at least one identical allele for each of the three markers in the pre-treatment and post-treatment samples. New infections were diagnosed when all alleles for at least one of the markers differed between the two samples. Cases with new infections were censored at the time of occurrence from the survival analyses. In case of failure after day 7, patients whose PCR results were unknown were censored at the time of occurrence from the survival analyses as well. A nested PCR was used to amplify the Kelch gene on chromosome 13 following previously reported methods [25, 26].

#### Classification standards for treatment outcome

Treatment outcome was categorized based on the WHO definitions for early treatment failure (ETF), late clinical failure (LCF), late parasitological failure (LPF), and adequate clinical and parasitological response (ACPR) [21].

### Statistical analyses

Data were double entered and cleaned in EpiData 3.1 (EpiData Association, Odense, Denmark), and analyzed in Epi Info 7 (Centers for Disease Control and Prevention, Atlanta, GA). Attack rate (AR) and incidence density (ID) was calculated using the following formula [27]:

$$ \mathrm{A}\mathrm{R}=\frac{\mathrm{Number}\;\mathrm{of}\kern0.37em \mathrm{persons}\kern0.24em \mathrm{attacked}}{\mathrm{Number}\ \mathrm{of}\ \mathrm{persons}\ \mathrm{investigated}}\times 100\% $$

$$ \mathrm{ID}=\frac{\mathrm{Total}\;\mathrm{number}\ \mathrm{of}\ \mathrm{attacks}}{\mathrm{Number}\ \mathrm{of}\ \mathrm{persons}\ \mathrm{investigated} \times \mathrm{number}\ \mathrm{of}\ \mathrm{weeks}\ \mathrm{observed}}\times 100 $$

A Chi-squared test was used to compare the proportions and significance was *P* < 0.05. A multivariate logistic model was used to identify risk factors of malaria infections during the outbreak. In the model, the outcome (dependent) variable was the RDT result, and independent variables were demographic characteristics, staying overnight in other places, seeking treatment, ownership of bed net and type of roof [27, 28]. The Kaplan-Meier method was used to analyze the outcome of therapeutic efficacy test of DP [18, 19, 21, 22].

### Ethical approval

The outbreak investigation and control protocol was approved by Wa State Department of Health (WDH). Oral agreements for the outbreak surveys and control interventions were obtained from all individuals. Ethical approval for in vivo efficacy study was granted by the Ethics Committee of Yunnan Institute of Parasitic Diseases (YIPD), China. Informed written consent was obtained from all adult subjects or caretakers of children who participated in DP therapeutic efficacy study, and the participants had the right to withdraw from the study at any time. All results were kept confidential and were unlinked to any identifying information.

## Results

### Outbreak characteristics

The outbreak was reported when three out of four patients were found to be positive for *P. falciparum* by microscopy. In order to better understand the focus of the outbreak further 50 inhabitants were tested by RDT and 32 (64 %, 95 % *CI*: 49.2–77.1 %) were found positive. This result prompted a formal outbreak investigation.

Of the 122 inhabitants of ARP, 92 were investigated. Two deaths were reported by the private healer and interviewees during the face-to-face interview. 90 (73.8 %) suspected cases were tested with both RDT and microscopy. Questionnaires were administered in all 90 subjects (Table 1). The four initially tested cases and the 50 tested with RDT by HPA were reexamined by microscopy and administered with questionnaires, and thus were included in the 90 total subjects to complete the questionnaire.

**Table 1:** Baseline characteristics of subjects in the outbreak site and its six surrounding communities, Shan Special Region II, Myanmar

Characteristics	Number (%, 95 % *CI*)
Subjects in ARP (*n* = 92)	
Male	44 (47.8, 37.3–58.5)
Age < 5 years	12 (13.0, 6.9–21.7)
Age 5–14 years	20 (21.7, 13.8–31.6)
Age ≥15 years	60 (65.2, 54.6–74.9)
Overnight in other places, May 1-June 19, 2014	12 (13.0, 6.9–21.7)
Seek treatment from the private healer	59 (64.1, 53.5–73.9)
Subjects with RDT positivity in ARP (*n* = 65)	
Male	30 (46.2, 33.7–59.0)
Age < 5 years	12 (18.5, 9.9–30.0)
Age 5–14 years	15 (23.1, 13.5–35.2)
Age ≥15 years	38 (58.5, 45.6–70.6)
Overnight in other places, May 1-June 19, 2014	8 (12.3, 5.5–22.8)
Seek treatment from the private healer	49 (75.4, 63.1–85.2)
Subjects with parasites in ARP (*n* = 38)	
Male	20 (52.6, 35.8–69.0)
Age < 5 years	8 (21.1, 9.6–37.3)
Age 5–14 years	10 (26.3, 13.4–43.1)
Age ≥15 years	20 (52.6, 35.8–69.0)
Overnight in other places, May 1-June 19, 2014	5 (13.2, 4.4–28.1)
Seek treatment from the private healer	20 (52.6, 35.8–69.0)
Axillary temperature (°C), Mean ± SD (rang)	37.1 ± 0.8 (36.1–39.2)
Parasite count (per uL), Geometric mean (rang)	24268 (860–99667)
Subjects in six surrounding communities (*n* = 328)	
Male	144 (43.9, 38.5–49.5)
Age < 5 years	46 (14.0, 10.5–18.3)
Age 5–14 years	95 (29.0, 24.1–34.2)
Age ≥15 years	187 (57.0, 51.5–62.4)
Overnight in other places, May 1-June 19, 2014	0 (0, 0–1.1)
RDT positivity	4 (1.2, 0.3–3.1)
*P. falciparum*	2 (0.6, 0.1–2.0)

The median age was 22.9 years (rang: 3 months-70 years). The male/female sex ratio was 0.96 (Table 1).

As a result of the retrospective data collection, the first malaria patient, a 52-year male, was diagnosed by the private healer on May 17 and died on May 27. He had been mining for gold in Salween River Valley and returned back to ARP on May 8; the second fatality, 23-year male, occurred on June 14, but he had never been out of ARP. The both fatalities were Lahu ethnic minority, and they presented the symptoms of complicated malaria including shivering, high fever, severe headache, hematuria, abnormal posturing, nystagmus and coma.

Out of the 90 suspected cases tested by RDT and microscopy, 65 (72.2 %) were positive for *P. falciparum* by RDT and 38 (42.2 %) were found positive by microscopy (*x*^2^ = 14.91, *P* < 0.01). From the 65 RDT positive subjects, 30 (46.2 %) were male and 27 (41.6 %) children (<15 years) (Table 1).

To estimate the size of the outbreak, a total of 328 volunteer participants from the six surrounding communities were screened by both RDT and microscopy in a short cross–sectional survey. The median age of the subjects was 21.0 years (rang: 1 month - 80 years). The male/female sex ratio was 0.8. Four (1.2 %) individuals were found positive for *P. falciparum* by RDT, three of them were from Banbai village, which is approximately 1 kilometer away from the centre of the outbreak. The fourth case was from Laodaoban village, which is two kilometers away from ARP. All 328 subjects did not report being overnight in other places from May 1^st^ to June 19^th^.

### Risk factors

Data to determine risk factors was collected in structured questionnaires from 90 participants. A multivariate logistic regression analysis (MLRA) identified seeking treatment from the private healer (*OR* 3.78, *P* < 0.01) and lack of bed nets (*OR* 3.21, *P* < 0.05) were associated with malaria infection during the outbreak. The most important risk factor was age <15 years with an OR of 13.5 (*P* < 0.01) (Table 2). Data collected in the unstructured interviews confirmed these findings. Many respondents reported that the private healer was their first choice when they were ill. The private healer just administered a single artemether injection as treatment. The ill persons also said that they did not go for more injections when they felt well unless they had another attack, resulting in a less-than-three-day artemether regimen (a standard artemether regimen for malaria treatment should be 5 days). The respondents also reported that no health staff had visited ARP until the outbreak, and they had never received bed nets prior.

**Table 2:** Results of multivariate logistic regression analysis for risk factors during the outbreak, Aidao Rubber Plantation, Shan Special Region II of Myanmar (*N* = 90)

	*P. falciparum* (%, 95 % *CI*)	RDT positivity (%, 95 % *CI*)	*OR* (95 % *CI*)	*P*-value	Adjusted *OR* (95 % *CI)*	*P*-value
Sex						
Female (*n* = 48)	20 (41.7, 27.6–56.8)	33 (68.8, 53.7–81.3)	1	-	1	-
Male (*n* = 42)	18 (42.9, 27.7–59.0)	32 (76.2, 60.5–87.9)	1.45 (0.52–4.12)	0.5821	1.27 (0.40–4.39)	0.6120
Age (years)						
46–74 (*n* = 11)	2 (18.1, 2.3–51.8)	3 (27.3, 6.0–61.0)	1	-	1	-
15–45 (*n* = 47)	18 (38.3, 24.5–53.6)	35 (74.5, 59.7–86.1)	7.78 (1.50–45.18)	0.0052	7.75 (1.48–44.97)	0.0049
0–14 (*n* = 32)	18 (56.3, 37.7–73.6)	27 (84.4, 67.2–94.7)	14.4 (2.27–107.82)	0.0010	13.51 (2.21–105.89)	0.0013
Overnight in other places from May 1 to June 19, 2014						
Yes (*n* = 12)	5 (15.2, 5.1–31.8)	8 (66.7, 34.9–90.1)	1	-	1	-
No (*n* = 78)	33 (42.3, 31.2–54.0)	57 (73.1, 61.8–82.5)	1.36 (0.30–5.74)	0.7316	1.08 (0.25–47.32)	0.8154
Seeking treatment from the private healer within 10 days						
No (*n* = 31)	18 (58.1, 39.1–73.6)	16 (51.6, 33.1–69.8)	1	-	1	-
Yes (*n* = 59)	20 (33.9, 22.1–47.4)	49 (83.2, 71.0–91.6)	4.59 (1.55–13.77)	0.0035	3.78 (1.30–46.18)	0.0045
Ownership of bed nets						
Yes(*n* = 16)	4 (25.0, 7.3–52.4)	7 (43.8, 19.8–70.9)	1	-	1	-
No(*n* = 74)	34 (45.9, 34.3–57.9)	58 (78.4, 67.3–87.2)	4.66 (1.31–16.80)	0.0125	3.21 (1.21–15.19)	0.0157
Type of roof						
Asbestos tile (*n* = 13)	3 (23.1, 5.0–53.8)	8 (61.5, 31.6–86.1)	1		1	-
Grass (*n* = 77)	35 (45.5, 34.1–57.2)	57 (74.0, 63.3–82.9)	1.78 (0.44–7.01)	0.3400	1.59 (0.50–9.18)	0.4152

Note: RDT positivity is the outcome (dependent) variable

### Attack rate and results of outbreak response and control

**Fig. 3:**
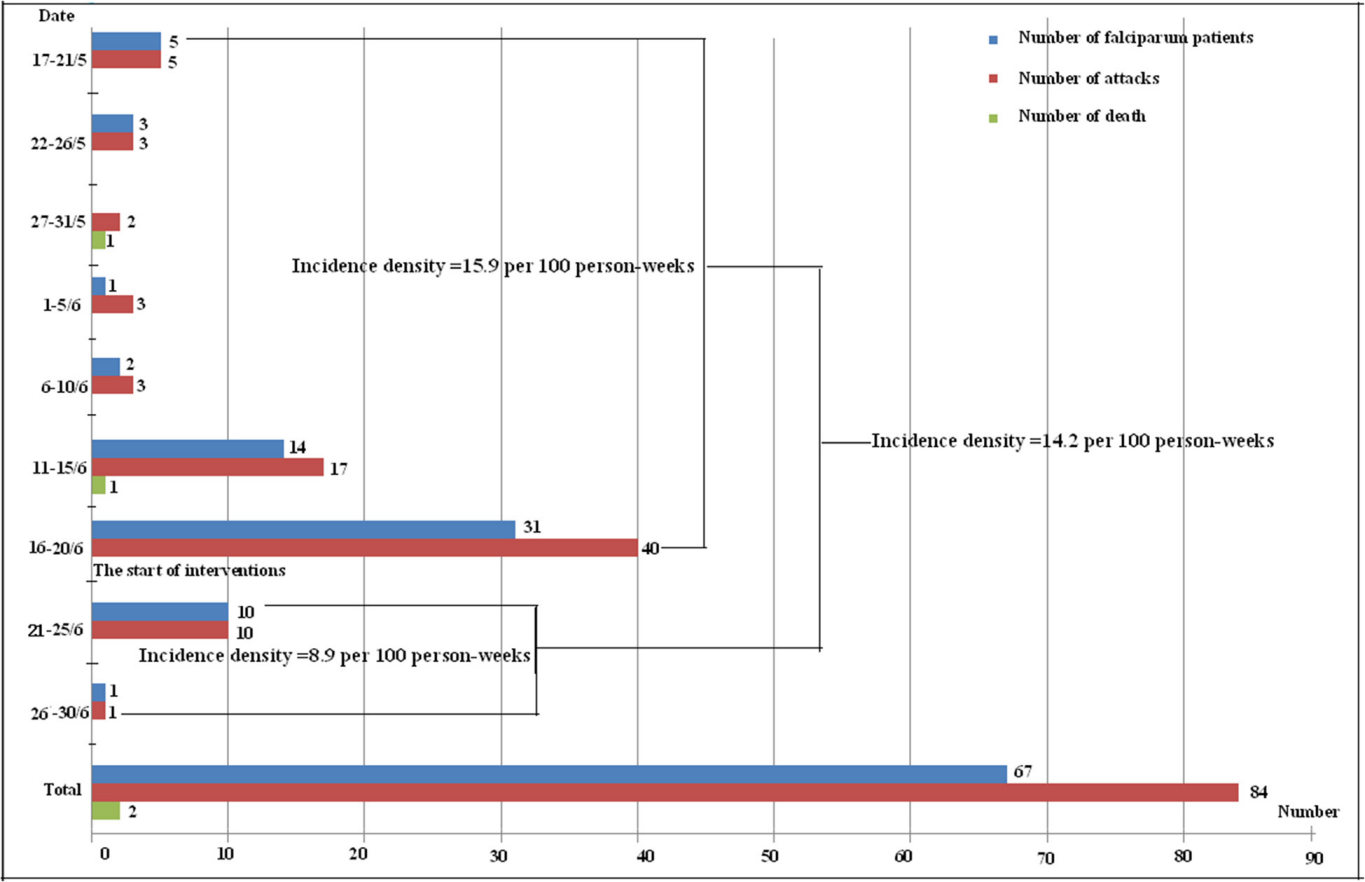
Incidence Chart of falciparum malaria (number of patients, attacks and death) for the outbreak in Shan Special Region II of Myanmar, June – December 2014

During the outbreak, prior to the first intervention, the attack rate and incidence density were 60.7 % (56/92) and 15.9 per 100 person-weeks respectively. After the intervention this fell to 12.2 % (11/90) and 8.9 per 100 person-weeks (Fig. 3). The response decreased the incidence density significantly (*χ*^2^ = 3.99, *P* < 0.05) and rapidly, within 10 days. There were no malaria attacks found after July 1^st^. The trained CHWs were unable to find any individuals with parasites and/or that were RDT positivity in ARP and the six surrounding communities. Furthermore, malaria has not been found in those communities until December 31^st^ 2014.

### Entomological results

From 8:00 pm of June 20^th^ to 7:00 am of June 21^st^ (11 h), 110 of anopheline mosquitoes were collected. The light traps collected nine anopheline mosquitoes indoors including one (11.1 %) *An. minimus* and four (44.4 %) *An. sinensis*, and 101 anopheline mosquitoes outdoors including eight (7.9 %) *An. minimus*, 20 (19.8 %) *An. maculates* and 20 *An. sinensis* (Table 3)*.*

**Table 3:** Results of entomological investigation in the outbreak site, Aidao Rubber Plantation, Shan Special Region II of Myanmar

Density (per lamp-night)		% (% *CI*)
Indoor (*n* = 9)		
*An. minimus*	1	11.1 (0.3–48.2)
*An. annularis*	4	44.4 (13.7–78.8)
*An. sinensis*	4	44.4 (13.7–78.8)
Outdoor (*n* = 101)		
*An. minimus*	8	7.9 (3.5–15.0)
*An. annularis*	50	49.5 (39.4–59.6)
*An. maculatus*	20	19.8 (12.5–28.9)
*An. splendidus*	3	3.0 (0.6–78.8)
*An. sinensis*	20	19.8 (12.5–28.9)

### Therapeutic efficacy of DAPQ

A total of 35 of 38 confirmed *P. falciparum* patients were recruited into the embedded DAPQ therapeutic efficacy trial. There were a total of two treatment failures, one LCF and one LPF. No patients were lost to follow up. The cure rate at day 42 was therefore 94.3 %, (95 % *CI*, 79–98.5 %) (Table 4. PCR identified the LCF and LPF as recrudescence on day 14 and 21 respectively, and the PCR-adjusted cure rate is therefore the same as the unadjusted. Both patients received a second course of DAPQ and were followed up further, without detecting parasites anymore. The fever clearance time (FCT) and asexual parasite clearance time (APCT) were, respectively, 36.4 ± 8.9 and 53.3 ± 11.3 h. K13-propeller mutations were not detected in these parasite samples.

**Table 4:** Therapeutic responses of patients to dihydroartemisinin-piperaquine, Shan Special Region II, Myanmar (*n* = 35)

Indicator	Value
Fever clearance time (hr), Mean (± SD)	36.4 (8.9)
50 % asexual parasite clearance time (hr), Mean (± SD)	25.2 (7.8)
Asexual Parasite clearance time (hr), Mean (± SD)	53.3 (11.3)
Early treatment failure, number (%, 95 % *CI*)	0 (0,0–6.7)
Late clinical failure, number (%, 95 % *CI*)	1 (2.9, 0.07–14.9)
Late parasitological failure, number (%, 95 % *CI*)	1 (2.9, 0.07–14.9)
Adequate clinical and parasitological response, number (%, 95 % *CI*)	33 (94.3, 79.0–98.5)

## Discussion

The purpose of the paper is to present the outbreak investigation, the public health response, DAPQ sensitivity in *P. falciparun*, cause analysis of the outbreak and implications of the findings.

According to the report from the private healer and the face-to-face interview, the gold miner was the first patient, and he died on May 27^th^. The interviews also found that patients usually visited the private healer and received less than 3-day artemether injection. The results of the parasitological investigation showed that the RDT positive rate (72.2 %) was much higher than the microscopically-determined parasite rate (38.9 %). As all slides were reexamined by two expert microscopists in YIPD and the RDT (SD FK80 P.f/P.v Malaria Antigen Rapid Diagnostic Test) detects histidine-rich protein-2 (HRP2) of *P. falciparum* and specific lactate dehydrogenase (Pv-pLDH) of *P. vivax* [29], one explanation for the discordance between detection methods is the private healer did not follow the required treatment course, and administered less than 3-day artemether injection. This sub-dosing of artemether decreased the *P. falciparum* parasitemia to sub-microscopy levels, but did not clear parasites and interrupt transmission completely. In addition, HRP2 is detectable by RDT for about 2 weeks after parasite clearance [30]. As the HRP2 in some patients treated with artemether was still at detectable levels by RDT when the investigation was conducted, the RDT positive rate might indicate a more accurate measure of the true prevalence of *P. falciparum* infections.

The PCR identified that all 35 parasite samples belonged to the same msp1, msp 2 and glurp genotype. The entomological survey found *An. minimus and An. sinensis* indoors; and a certain proportion of *An. maculates, An. sinensis* and *An. minimus* outdoors. *An. minimus* is considered the local principle vector; *An. sinensis* is a secondary vector and *An. maculates* is an important vector in the GMS [20]. These results show that the gold miner introduced *P. falciparum* to the existing vectors, and that the existing vectors were able to reestablish transmission.

The multivariate logistic regression analysis (MVLRA) identified that age, seeking inappropriate treatment and lack of bed nets were risk factors during the outbreak. Children less than 5 years accounted for 32.3 % of total 65 RDT positive subjects. This indicated that children, especially less than 5 years, were at the highest risk during the outbreak. While the sixth and tenth rounds of the GFATM’s China Malaria Program covered the Shan Special Region II of Myanmar, the malaria control programs neglected the ARP. As the ARP was a unique community consisting of the illegal, Chinese-immigrant Lahu people and the internal, Myanmar-immigrant Wa people, the inhabitants were too poor to seek appropriate diagnosis and effective treatment. Their first choice was to visit the private healer when they were ill. The private healer incorrectly administered a single artemether injection for malaria treatment and the ARP inhabitants did not return for the additional injections if they felt well. This led to artemether injection for less than 3 days. This sub-therapeutic dose of artemether relieved symptoms of malaria patients, but allowed residual parasites to maintain transmission and establish the outbreak. Additionally, the malaria control programs routinely distributed LLINs resulting in one net per two persons, however, the ARP had never received any LLINs prior to the outbreak, thus preventing protection from the mosquito vector.

The life cycle of *P. falciparum* is assumed to last about 10 days [31]. In this case, the peak of incidence from June 16^th^ to 20^th^ would indicate the third generation of patients from the initial outbreak (Fig. 3). As one would expect, the last patient to contract the disease appeared on June 30, i.e. 10 days after the emergence response. This showed the response was both timely and effective. According to the Chinese Ministry of Health’s strategy for approaching malaria outbreaks, mass drug administration (MDA) should be targeted to communities with recent outbreaks [31]. DAPQ was only administered to individuals with a positive RDT, however, two trained CHWs were equipped with RDT and DP, and conducted daily visits to the outbreak site and its surrounding communities. These CHWs conducted active detection and treated RDT positive patients immediately. Meanwhile, LLIN, IRS and BCC interventions were conducted in parallel. These control measures were effective as *P. falciparum* was just detected in two individuals, and there were only four positive RDTs in the six surrounding communities after the interventions. The strategy, with the noted absence of out MDA, had controlled the outbreak and prevented *P. falciparum* from spreading in the six surrounding communities. This success is likely attributed to active collaboration and joint efforts of China, Myanmar and international NGOs as well as strong community involvement.

DAPQ was the most commonly used ACT in malaria programs on the China-Myanmar border. While artemisinin resistance has extended across much of Myanmar [26], the results of the embedded efficacy study showed that the *P. falciparum* in this outbreak was still sensitive to DAPQ and that DAPQ was effective for the treatment of this outbreak, noting the absence of K13-propeller mutations in these parasite samples. Additional to this observation, the results of in vivo monitoring from 2007 to 2013 that indicate that *P. falciparum* was sensitive to DAPQ along China-Myanmar border [19], demonstrate the ongoing success of China’s strategy to control cross-border transmission of malaria and contain artemisinin resistance. However, this outbreak warns that malaria may resurge at any time as long as control activities weaken. Inappropriate treatments with sub-therapeutic-dosage and/or mono-therapies contribute to maintain malaria transmission and are thus harmful to patient prognosis and public health and also a cause of drug resistance [9]. While the emergence of artemisinin resistance in Southeast Asia is threatening the global control of *P. falciparum* malaria [25], these inappropriate treatments threaten to undermine usefulness of ACTs in areas that are unaffected by resistance.

Several lessons can be learned from the outbreak. First, particular attention should be given the transient and neglected groups. The lack of effective interventions and surveillance among neglected populations may lead to outbreaks or even a resurgence of malaria. Second, inappropriate diagnoses and treatments still exist in the private sector on the China-Myanmar border. Coverage and service of public health facilities should be increased and strengthened to allow access to easy alternative to private healers. Additionally, the ban of sale of fake and sub-standard antimalarial drugs, including artemisinin monotherapy should be more thoroughly enforced. Third, as the GFATM terminated the tenth round of its malaria program by December 31^st^, 2013, the outbreak occurred during the transition phase from China’s to Myanmar’s malaria program when surveillance and interventions were the weakest. Taken together, these lessons show that collaboration between China, Myanmar and international societies for malaria control should be strengthened and improved.

It is noted that the entomological survey is one limitation of his study as the collection of anopheline mosquitoes was only carried out by two light traps for one night, and human landing collection was not carried out. Entomological data of local malaria vectors are still lacking in Shan Special region II of Myanmar, and further research may be needed. However, the light traps were successful in collecting local malaria vectors including: *An. minimus*, *An. maculate* and *An. sinensis*. This provided evidence for inclusion of LLINs and IRS as part of outbreak response.

## Conclusion

This investigation confirmed, contained, and characterized *P. falciparum* malaria outbreak. Age, seeking inappropriate treatment and lack of bed nets were risk factors of malaria infection during the outbreak. K13-propeller mutations were not found in the *P. falciparum* parasites and DAPQ was demonstrably effective in treating infections. Treatment of malaria cases, combined with LLIN distribution, IRS and BCC, successfully controlled the outbreak and prevented the spread of *P. falciparum*. On the China-Myanmar border, malaria control, especially among special populations, needs further collaboration between China, Myanmar and international societies.

## Additional file

Additional file 1: Multilingual abstracts in the six official working languages of the United Nations. (PDF 415 kb)
