# Supplementary material for: Investigation and control of a Plasmodium falciparum malaria outbreak in Shan Special Region II of Myanmar along the China-Myanmar Border from June to December 2014
Source: Infect Dis Poverty. 2016 Apr 25;5:32. doi: 10.1186/s40249-016-0127-8 (PMC4843199; doi:10.1186/s40249-016-0127-8)
Supplement: Additional file 1: — Multilingual abstracts in the six official working languages of the United Nations. (PDF 415 kb) [file 40249_2016_127_MOESM1_ESM.pdf]

Translation of the abstract into the five official working languages of the United Nations

البحث والسيطرة على تفشي الملاريا *المتصورة المنجلية* في منطقة شان الخاصة 2 في ميانمار على طول الحدود بين الصين وميانمار من يونيو إلى ديسمبر 2014

Hui Liu, Jian-wei Xu, Heng-lin Yang, Mei Li, Cheng-de Sun, Yi-jie Yin, Zhi-liang Zheng, Guang-yun Zhang, Ai-shui Yu, Yong-hui Yang, Chun-hui Li, Shui Ai

الملخص

الخلفية: منذ 2007 وحتى 2013، أدت التدابير الصارمة لمكافحة الملاريا إلى انخفاض معدلات الإصابة بهذا المرض بنسبة 90% على طول الحدود بين الصين وميانمار. ورغم هذه التدابير، فقد انتشرت الملاريا المتصورة المنجلية في منطقة شان الخاصة 2 في ميانمار في يونيو 2014.

المنهجية: أجريت تحقيقات حول الوباء والطفيليات والحشرات، واستخدم"Dihydroartemisinin piperaquine" على الفور لعلاج الأفراد المصابين بالطفيليات، كما استخدمت شبكات الحشرات الدائمة والرش الموضعي داخل المباني واتبعت سلوكات وأنماط اتصال مختلفة للسيطرة على المرض المتفشي. وأجريت دراسة فعلية وضمنية لتقييم "Dihydroartemisinin piperaquine"، وطُبّق التنميط الجيني الجزيئي عن طريق تفاعل البوليميزير المتسلسل على الجين "Kelch" على الكروموسوم 13.

النتائج: تم تحديد جميع حالات العدوى على أنها بسبب الطفيليات المتصورة المنجلية من خلال اختبارات التشخيص السريع والفحص المجهري. سُجلت حالتا وفاة ناجمتان عن تفشي المرض. بلغ معدل الهجوم نسبة 72.8% (92/67) كما بلغ معدل الإصابة 14.2 لكل 100 شخص-أسابيع. وتم تحديد 72.2% (90/65) من خلال اختبارات التشخيص السريع و42.2% (90/38) من خلال الفحص المجهري لتحديد الفطريات. نسبة الاحتمالات المعدَّلة لتحليل الانحدار اللوجستي متعدد المتغيرات لمن يبلغ <15 عامًا، و15-45 عامًا، والعلاج غير المناسب من قبل معالج خاص وكذلك الافتقار إلى الناموسيات 13.51 (95% حدود الثقة، 2.21-105.89)، 7.75 (1.48-44.97)، 3.78 (1.30-46.18) و 3.21 (1.21-15.19) على التوالي. وفي المجتمعات الستة المحيطة بمنطقة تفشي المرض، بلغت نسبة الإصابات التي تم التعرف عليها من خلال اختبارات التشخيص السريع 1.2% (328/4)، و 0.6% (328/2) التي تم التعرف عليها من خلال الفحص المجهري لتحديد الطفيليات. جُمعت 110 بعوضات من نوع أنوفيليس بما فيها الناقلات المحلية (*An. minimus, An. sinensis and An. maculates*) في مصيدتين ضوئيتين. وبعد اتباع تدابير صارمة للسيطرة عليها، انخفض عدد هجمات الملاريا والطفيليات ومولدات المضادات إلى الصفر بين 1 يوليو و1 ديسمبر 2014. بلغ معدل الشفاء لدى المرضى المصابين بالملاريا المتصورة المنجلية في اليوم 42 نسبة 94.3% (95% CI، 80.8-99.3%). ولم يكشف تفاعل البوليميراز المتسلسل عن طفرات في جين K13-propeller

الخلاصة: أدت طفيليات متصورة منجلية وافدة إلى تفشي هذا المرض. يعد السن، والعلاج غير المناسب، والافتقار إلى الناموسيات من عوامل الخطر التي تؤدي إلى العدوى خلال تفشي المرض. أظهرت الطفيليات المتصورة المنجلية استجابة حساسة للعلاج باستخدام "Dihydroartemisinin piperaquine". ساهمت التدابير المتكاملة في السيطرة على تفشي المرض وحالت دون انتشار الطفيليات المتصورة المنجلية على نحو فعال. تشير نتائج هذه الدراسة إلى أن السيطرة على الملاريا على الحدود بين الصين وميانمار، وبين السكان على وجه الخصوص، تتطلب المزيد من التعاون بين الصين وميانمار والمجتمعات الدولية.

Translated from English version into Arabic by Shada Salameh, through

TRANSLATORS WITHOUT BORDERS

中缅边境缅甸掸邦第二特区 2014 年 6-12 月恶性疟暴发调查和控制

Hui Liu, Jian-wei Xu, Heng-lin Yang, Mei Li, Cheng-de Sun, Yi-jie Yin, Zhi-liang Zheng, Guang-yun Zhang, Ai-shuiYu, Yong-hui Yang, Chun-hui Li, Shui Ai

摘要
引言: 经强化控制，中缅边境疟疾负担 2007-2013 间减少了 90%，然而缅甸掸邦第二特区于 2014 年 6 月报告了一起恶性疟暴发流行。
方法: 开展了流行病学、寄生虫病学和昆虫学调查。对寄生虫检测阳性者立即给予科泰复治疗，发放长效蚊帐(LLIN)，开展杀虫剂室内滞留喷洒(IRS)和行为改变的交流。同时，开展科泰复临床治疗效果评价，并采用 PCR 检测 K13 基因。
结果: RDT 和镜检确认全部感染均为恶性疟原虫，2 人死亡，发作率 72.8% (67/92)，周发病率 14.2%。 RDT 阳性率 72.2% (65/90)，镜检阳性率 42.2% (38/90)。经多因素 logistic 回归分析，年龄<15 岁, 15-45 岁，到个体行医者处寻求治疗和缺乏蚊帐调整后的比值比（OR）分别是 13.51 (95%可信区间：2.21-105.89), 7.75 (1.48-44.97), 3.78 (1.30-46.18) 和 3.21(1.21-15.19)。暴发点周围 6 个社区 RDT 阳性率为 1.2% (4/328)，镜检原虫阳性率 0.6%(2/328)。两盏诱蚊灯在暴发点一个晚上捕到 110 只按汶，包括有当地疟疾媒介微小按汶、中华按汶和多斑按蚊。实施防治措施后，2014 年 7-12 月疟疾发作，镜检到疟原虫和 RDT 检测到抗原数均为零，随访 42 天科泰复的恶性疟病人治愈率 94.3%(95% CI, 80.8-99.3%)，PCR 未检测到 K13 基因突变。
结论： 输入性恶性疟引起了本次暴发。年龄、不恰当求医行为和缺少蚊帐是这次暴发期间疟疾感染危险因素。恶性疟原虫对科泰复仍然敏感。综合性措施有效地控制了这次暴发，防止了恶性疟原虫扩散。研究结果显示中缅边境，特别是特殊人群中的疟疾控制，需要中缅双方和国际社会间的进一步合作。

Translated from English version into Chinese by Xu Jianwei

Examen et lutte contre une épidémie de paludisme à *Plasmodium falciparum* dans la région spéciale II de l’État Shan au Myanmar le long de la frontière sino-birmane de juin à décembre 2014

Hui Liu, Jian-wei Xu, Heng-lin Yang, Mei Li, Cheng-de Sun, Yi-jie Yin, Zhi-liang Zheng, Guang-yun Zhang, Ai-shuiYu, Yong-hui Yang, Chun-hui Li, Shui Ai

Résumé
Contexte : de 2007 à 2013, des mesures de lutte intensives ont permis de réduire le fardeau du paludisme de 90 % le long de la frontière sino-birmane. Une épidémie de paludisme à *P. falciparum* a néanmoins été signalée malgré ces efforts dans la région spéciale II de l’État de Shan au Myanmar en juin 2014.
Méthodes : des examens épidémiologiques, parasitologiques et entomologiques ont été réalisés. De la dihydroartémisinine-pipéraquine (DHA-P) a été immédiatement administrée pour traiter les individus porteurs du parasite. Des moustiquaires imprégnées d’insecticide de longue durée, la pulvérisation d’insecticides à effet rémanent à l’intérieur des habitations et les mesures de communication destinées à la promotion du changement de comportement ont aussi été fournis pour lutter contre l’épidémie. Une étude d’efficacité intégrée a été menée pour évaluer l’administration de DHA-P. Le génotypage moléculaire par réaction en chaîne par polymérase (PCR) a été réalisé sur le gène Kelch sur le chromosome 13.
Résultats : toutes les infections ont été identifiées comme étant à *Plasmodium falciparum* par l’intermédiaire d’un test de diagnostic rapide et un examen microscopique. Cette épidémie a provoqué deux décès. Le taux de crise atteignait 72,8 % (67/92) et le taux de densité d’incidence s’élevait à 14,2 pour 100 personnes-semaines. Le taux positif du test de diagnostic rapide atteignait 72,2 % (65/90) et le taux de présence de parasites déterminée par microscopie s’élevait à 42,2 % (38/90). Le rapport de cotes ajusté (OR) de l’analyse de régression logistique multivariée de personnes < 15 ans, de personnes âgées de 15 à 45 ans, du traitement inapproprié par un guérisseur et du manque de moustiquaires atteignait respectivement 13,51 (intervalle de confiance à 95 %, 2,21-105,89), 7,75

(1,48-44,97), 3,78 (1,30-46,18) et 3,21(1,21-15,19). Dans les six communautés environnant le site d'épidémie, le taux positif du test de diagnostic rapide atteignait 1,2 % (4/328) et le taux de présence de parasites déterminée par microscopie s'élevait à 0,6 % (2/328). Deux pièges lumineux ont permis de collecter au total 110 moustiques anophèles, dont des vecteurs locaux, *An. minimus*, *An. sinensis* et *An. maculates*. Après une lutte intensive, la détection de crises de paludisme, de parasites et d'antigènes a été réduite à zéro entre le 1er juillet et le 1er décembre 2014. Le taux de guérison de patients porteurs du *P. falciparum* au jour 42 atteignait 94,3 % (IC à 95 %, 80,8-99,3 %). La PCR n'a pas permis de détecter des mutations au sein du domaine en hélice du gène K13.

Conclusion : le *P. falciparum* importé est à l'origine de l'épidémie. L'âge, le recours à un traitement inapproprié et le manque de moustiquaires constituaient des facteurs de risque d'infection au cours de l'épidémie. Le *P. falciparum* était sensible au traitement par DHA-P. Les mesures intégrées ont permis de lutter contre et de prévenir efficacement l'épidémie de *P. falciparum*. Les résultats de cette étude indiquent que la lutte contre le paludisme à la frontière sino-birmane, notamment parmi des populations spéciales, nécessite un approfondissement de la collaboration entre la Chine, le Myanmar et les organismes internationaux.

Translated from English version into French by eric ragu, through

TRANSLATORS
WITHOUT BORDERS

Исследование и контроль вспышек малярии (возбудитель – паразитический микроорганизм *Plasmodium falciparum*) проводились в Особом регионе №2 штата Шан, вдоль Китайско-Мьянманской границы с июня по декабрь 2014 года.

Лю Хуэй, Хуан Вэй Жу, Хэн Линь Ян, Мэй Ли, Чэн Дэ Сунь, И Цзе Инь, Чжи Лян Чжэн, Гуан Юнь Чжан, Ай Шуй Юй, Юн Хуэй (Хой) Ян, Чунь Хуэй (Хой) Ли, Шуй Ай

Резюме

Общие сведения: С 2007 по 2013 год, благодаря усиленным мерам контроля, уровень заболеваемости мялярией вдоль Китайско-Мьянманской границы сократился на 90%. Однако, несмотря на эти меры, в июне 2014 года сообщалось о вспышке малярии (возбудитель – *P. falciparum*) в Особом регионе №2 штата Шан в Мьянме.

Методы: Были проведены эпидемиологические, паразитологические и энтомологические исследования. Для лечения инфицированных паразитами людей был незамедлительно применен дигидроартемизинин пиперахин (ДП). Также, для борьбы с эпидемическими заболеваниями были предоставлены противомоскитные сетки длительного использования, обработанные инсектицидами, распылители инсектицидов остаточного действия для использования внутри помещений и информационная поддержка изменения поведения. Было проведено подисследование эффективности применения дигидроартемизинина пиперахина (ДП). Ген *Kelch*, расположенный на хромосоме 13, был подвергнут молекулярному генотипированию при помощи полимеразной цепной реакции (ПЦР).

Результаты: Посредством микроскопии и быстрых диагностических тестов (БДТ) было выявлено, что возбудителем всех инфекционных заболеваний стал Plasmodium falciparum. В результате вспышки эпидемии погибли два человека. Частота поражений составила 72,8% (67/92), а плотность заболеваемости

– 14,2 на 100 человеко-недель. Быстрые диагностические тесты (БДТ) дали положительный результат в 72,2% (65/90) случаев, а микроскопически определяемый паразитарный индекс составил 42,2% (38/90). Скорректированное отношение шансов (ОШ) многофакторного регрессионного анализа для таких факторов, как попадание в возрастную группу до 15 лет, 15-45 лет, неподобающее лечение у народных целителей, а также отсутствие надкроватных сеток, обработанных инсектицидами, составило 13,51 (95% доверительного интервала, 2,21-105,89), 7,75 (1,48-44,97), 3,78 (1,30-46,18) и 3,21 (1,21-15,19) соответственно. В шести близлежащих районах вокруг места вспышки болезни, проведенные быстрые диагностические тесты (БДТ) дали положительный результат в 1,2% (4/328) случаев, а микроскопически определяемый паразитарный индекс составил 0,6% (2/328). Двумя световыми ловушками для насекомых в общей сложности удалось собрать 110 малярийных комаров, в число которых входят местные переносчики инфекции – малярийные комары рода *Anopheles*: *An. minimus, An. sinensis and An. maculates*. Усиленный контроль показал, что случаи выявления приступов малярии, паразитирующих организмов и антигенов сократились до нуля в период с 1 июля по 1 декабря 2014 года. Ежедневный показатель эффективности лечения больных малярией (возбудитель – *P. falciparum*) составил 94,3% (95% ДИ, 80,8-99,3%). Полимеразная цепная реакция (ПЦР) не выявила мутаций в «пропеллерном» домене белка, кодируемого геном K13.

Заключение: Завезенная малярия (вызываемая паразитическим микроорганизмом *P. falciparum*) стала причиной вспышки заболевания. Факторами риска инфицирования во время вспышки эпидемии являлись принадлежность к определенной возрастной группе, неправильное лечение, а также отсутствие надкроватных сеток, обработанных инсектицидами. Паразитические микроорганизмы *P. falciparum* чувствительны к лечению дигидроартемизинином пиперахином. Комплексные меры контролируют вспышки эпидемии и эффективно препятствуют распространению малярии (возбудитель – *P. falciparum*). Результаты данного исследования свидетельствуют о том, что продолжение борьбы с малярией на Китайско-Мьянманской границе, особенно среди отдельных групп населения, нуждается в дальнейшем укреплении сотрудничества между Китаем, Мьянмой и международным сообществом.

Translated from English version into Russian by Tatsiana Mankevich, through

TRANSLATORS
WITHOUT BORDERS

Investigación y control de un brote de malaria por *Plasmodium falciparum* en la Región Especial II en Shan, Myanmar en la frontera entre China y Myanmar entre junio y diciembre del año 2014

Hui Liu, Jian-wei Xu, Heng-lin Yang, Mei Li, Cheng-de Sun, Yi-jie Yin, Zhi-liang Zheng, Guang-yun Zhang, Ai-shuiYu, Yong-hui Yang, Chun-hui Li, Shui Ai

Resumen

Antecedentes: Entre el año 2007 y el año 2013, intensivas medidas de control lograron disminuir en un 90% la carga de la malaria en la frontera entre China y Myanmar. Sin embargo, a pesar de estas medidas, se reportó un brote de malaria por *P. falciparum* en la Región Especial II en Shan, Myanmar en junio de 2014.

Métodos: Se llevaron a cabo investigaciones epidemiológicas, parasitológicas y entomológicas. De inmediato se

administró dihidroartemisinina piperaquina (DP) para tratar el parásito en individuos positivos. También se proporcionaron redes con tratamiento insecticida de largo plazo (LLIN), rociado residual intradomiciliario (IRS) con insecticidas y comunicación para el cambio de comportamiento (BCC). Se llevó a cabo un estudio de eficacia integrado para evaluar la DP. Se llevó a cabo genotipificación molecular vía reacción en cadena de la polimerasa (PCR) sobre el gen Kelch del cromosoma 13.

Resultados: Mediante test de diagnostico rápido y microscopía se identificó a todas las infecciones como producidas por *Plasmodium falciparum*. Como consecuencia del brote se produjeron dos muertes. La tasa de ataque fue del 72,8% (67/92) y la tasa de incidencia o densidad fue del 14,2 por cada 100 persona-semanas. El índice positivo del test de diagnóstico rápido (RDT) fue del 72,7% (65/90) y la tasa de determinación del parásito por microscopía del 42,2% (38/90). La razón de momios (RM) ajustada del análisis de regresión logística multivariable para el grupo de edad de <15 años, 15-45 años, tratamiento inadecuado de un curador privado y la falta de redes para camas fueron del 13,51 (95% intervalo de confianza, 2,21-105,89), 7,75 (1,48-44,97), 3,78 (1,30-46,18) y 3,21(1,21-15,19) respectivamente. En las seis comunidades en los alrededores del sitio del brote, la tasa positiva del RDT fue del 1,2% (4/328) y la tasa de determinación del parásito por microscopía del 0,6% (2/328). Dos trampas de luz recolectaron un total de 110 mosquitos anopheles incluyendo vectores locales, *An. minimus*, *An. sinensis* y *An. maculates*. Luego de un control intensivo, la detección de ataques, parásitos y antígenos de malaria se redujo a cero entre el primero de julio y el primero de diciembre de 2014. El índice de cura de los pacientes con *P. falciparum* para el día 42 fue del 94,3% (95% IC, 80,8-99,3%). La PCR no detectó las mutaciones en la hélice del K13.

Conclusión: Un *P. falciparum* importado fue la causa del brote. Durante el brote, la edad, el tratamiento inadecuado y la falta de redes para las camas fueron factores de riesgo para la infección. El *P. falciparum* fue sensible al tratamiento con DP. Las medidas integradas controlaron el brote y previnieron la propagación efectiva del *P. falciparum*. Los resultados del presente estudio indican que el control de la malaria en la frontera entre China y Myanmar, en particular entre poblaciones especiales, necesita de mayor colaboración entre China, Myanmar y la sociedad internacional.

Translated from English version into Spanish by Maria Alejandra Aguada, through

TRANSLATORS WITHOUT BORDERS
